# The validity of the Czech version of Body Appreciation Scale-2 for adolescents

**DOI:** 10.1186/s40337-023-00897-7

**Published:** 2023-10-05

**Authors:** Nikol Kvardova, David Lacko, Hana Machackova

**Affiliations:** Interdisciplinary Research Team on Internet and Society, Faculty of Social Studies, Masaryk University, Jostova 10, 60200 Brno, Czech Republic

**Keywords:** Body appreciation Scale-2, Body appreciation, Body image, Validity, Adolescents, Czech Republic

## Abstract

**Background:**

Understanding the formation of body image is critical for the prevention and treatment of eating disorders, especially in adolescence, when body image develops significantly. One of the important facets of body image is body appreciation, which consists of positive feelings and attitudes towards the body regardless of its perceived “flaws”. To measure body appreciation, Body Appreciation Scale-2 (Tylka and Wood-Barcalow in Body Image 12:53–67, 2015a), a unidimensional 10-item measure, has been developed and routinely used in body image research. The current study examined the validity (i.e., factor structure, gender and age invariance, associations with other constructs) of the Czech version of Body Appreciation Scale-2 for adolescents.

**Methods:**

The study used two large samples of Czech adolescents, aged 13–18 (*N*_1_ = 613, *M* = 15.5, 52% girls; *N*_2_ = 1,530, *M* = 15.4, 50% girls). The data were collected in August 2021 (*N*_1_) and November 2020 (*N*_2_) through an online survey. For the data analysis, we used confirmatory factor analysis (CFA), multi-group confirmatory factor analysis (MG-CFA), and Structural Equation Modeling (SEM).

**Results:**

Our findings supported the proposed unidimensional factor structure and the gender (i.e., girls, boys) and age (i.e., 13–15, 16–18) scalar invariance of the Czech version of Body Appreciation Scale-2. The data also showed the expected positive correlations with body satisfaction and self-esteem, and negative correlations with media-ideal internalization, appearance schematicity, and depression. Furthermore, we discovered that body appreciation was more strongly connected to media-ideal internalization and depression for girls than boys.

**Conclusions:**

The present study provided robust evidence that supports the validity of the Czech version of Body Appreciation Scale-2 and its usability for the assessment of body appreciation in Czech adolescents. We also proposed future directions for the research on body appreciation based on the explored gender differences.

**Supplementary Information:**

The online version contains supplementary material available at 10.1186/s40337-023-00897-7.

## Abstract (plain language summary)

Understanding the formation of body image—that is, how people view and evaluate their bodies—is crucial for the prevention and treatment of eating disorders. This is especially true in adolescence, when body image develops significantly. That being said, the psychological assessment of body image, both in research and practice, requires reliable, high-quality measurement scales. Since its development by Tylka and Wood-Barcalow (2015a), Body Appreciation Scale-2 has been routinely used to assess body appreciation, which includes positive attitudes towards one’s body despite its perceived “flaws”. Our study provides evidence for the quality of the Czech version of Body Appreciation Scale-2 for use with adolescents. We examined the scale’s characteristics on data from two robust samples (613 and 1530 Czech adolescents). Our study showed that the qualities of Czech Body Appreciation Scale-2 are satisfactory, and we recommend the scale for the assessment of body appreciation for adolescents in the Czech context. We also documented that the scale scores can be accurately compared between adolescent girls and boys, and younger (13–15) and older (16–18) adolescents. Additionally, we discovered gender differences in the relationships between body appreciation and depressive moods and the internalization of media ideals, which demands further exploration in future research.

**Supplementary Information:**

The online version contains supplementary material available at 10.1186/s40337-023-00897-7.

## Background

Body appreciation consists of viewing one’s body positively, irrespective of actual appearance, including caring for what the body needs and resisting unattainable media ideals (Tylka 2019). The measurement of body appreciation is crucial in both research and practice, so Body Appreciation Scale-2 (BAS-2; Tylka and Wood-Barcalow 2015a) was developed and adapted to multiple languages, including Dutch (Alleva et al. 2016), Danish, Swedish, Portuguese (Lemoine et al. 2018), Polish (Razmus and Razmus 2017), Spanish (Swami et al. 2017), Romanian (Swami et al. 2017), Italian (Casale et al. 2021), Mandarin Chinese (Ma et al. 2022), German (Behrend and Warschburger 2022), Korean (Lee 2023), and others. The above-mentioned research has documented the scale’s validity, most recently in the adolescent population (Escoto Ponce de León et al., 2021; Góngora et al. 2020; Lemoine et al. 2018; Paquette et al. 2022).

Adolescence is a susceptible period for body image development: The changes in body weight and shape, the salience of peer influence, and increased self-awareness make adolescents attentive to their body appearance and increase the perceived importance of body image (Rodgers and Paxton 2014). Cultivating body appreciation as a manifestation of positive body image might shield adolescents from body dissatisfaction (e.g., Frisén and Homlqvist 2010; Webb et al. 2014), and likely from eating disturbances (e.g., Linardon et al. 2022), which emphasizes how critical the assessment of body appreciation is during adolescence.

The present study examined the validity of the BAS-2 in the population of Czech adolescents aged 13–18. Investigating the properties of the Czech version of the BAS-2 is warranted, because such evidence is lacking and it could encourage positive body image research in the Czech context. We investigated the BAS-2’s factor structure. Considering the age (e.g., Holsen et al. 2012) and gender (e.g., He et al. 2020) variations in adolescent body image, we examined the scale’s age and gender invariance. Also, previous research showed positive correlations between body appreciation and diverse well-being indicators (e.g., Avalos et al. 2005; Tylka and Wood-Barcalow 2015a; Webb et al. 2015). The current study added to this line of research and investigated the BAS-2’s relationship with body satisfaction, media-ideal internalization, self-esteem, depression, and appearance schematicity. Beyond the examination of the validity of the Czech BAS-2, we also explored gender and age differences in body appreciation, and gender differences in the relationships of body appreciation with the above-mentioned body image and well-being constructs, because these have not yet been studied extensively. Our study provides evidence for the usability of the Czech version of Body Appreciation Scale-2 and contributes to the knowledge of gender and age patterns in body appreciation in adolescence.

### Body appreciation in the multidimensional structure of body image

Body image reflects how people view their bodies. It consists of perceptive (e.g., perception of thinness), affective (e.g., appearance anxiety), cognitive (e.g., body-related cognitive distortions), and behavioral (e.g., body checking) dimensions (Delinsky 2011). Body image constructs can also be divided into two major groups: the “negative” perspective of body-related concerns (i.e., *negative body image*), which has dominated the body image field for decades, and the one that emerged within positive psychology (i.e., *positive body image*) (Tylka and Wood-Barcalow 2015a). Positive body image does not lie on the same continuum as negative body image: It is a qualitatively different dimension that goes beyond mere appearance satisfaction (Tylka and Wood-Barcalow 2015a). Wood-Barcalow et al. (2010) defined positive body image as: (a) the appreciation of the unique features and functions of the body; (b) the acceptance and admiration of the body; (c) feelings of beauty, confidence, and happiness with the body; (d) emphasis on the positive features of the body rather than dwelling on its “imperfections”; (e) a mindful connection to the needs of the body; and (f) the body-protective cognitive processing of information. Positive information is internalized and negative information is reframed or rejected. Body appreciation, measured by Body Appreciation Scale-2 (Tylka and Wood-Barcalow 2015a), reflects multiple body image dimensions, such as cognitive (e.g., attention to the body’s needs), emotional (e.g., feeling beautiful), and behavioral (e.g., acts revealing positive attitude towards the body), and it covers all of the aforementioned facets of positive body image. Body appreciation is defined as having positive opinions towards the body, regardless of the actual physical appearance, including accepting the body despite its perceived “imperfections”, respecting and caring for the body’s needs, engaging in healthy behaviors, and rejecting overly attractive appearance ideals (Tylka 2019). Body appreciation goes beyond liking one’s appearance or how it aligns with societal beauty ideals; rather, it involves cherishing the body for its unique features, for what it is able to do, and for what it represents (Tylka and Wood-Barcalow 2015b). In adolescence, Maes et al. (2021) further differentiate between body-self and body-other appreciation. Our study, as well as Body Appreciation Scale-2 (Tylka and Wood-Barcalow 2015a), follows the conceptualization of body appreciation in relation to one’s own body. Acting as a protective characteristic, even after accounting for other body image constructs, body appreciation is associated with lower body surveillance, less eating pathology, less negative affect, and fewer depressive symptoms; and, on the other hand, it is associated with higher body and functionality satisfaction, more body image flexibility, intuitive eating, self-compassion, and self-esteem (Linardon et al. 2022). Among adolescents, higher body appreciation predicts lower body dissatisfaction (Ren et al. 2023), a lower amount of dieting, improved intuitive eating, higher body acceptance by others, and improved mental well-being (Linardon et al. 2022), even longitudinally.

### Psychometric characteristics of Body Appreciation Scale-2

The first version of the Body Appreciation Scale (BAS) was developed by Avalos et al. (2005). The 13 items for the theoretical components of positive body image were answered on a scale from 1 (*Never*) to 5 (*Always*). The unidimensional factor model showed an adequate fit. The authors also documented its internal consistency (α = 0.94), test-retest reliability over a three-week period (*r* = .90), item-total correlations (> 0.46), and construct and incremental validity in a sample of college women. Although the scale was initially developed and tested on a sample of women, its gender invariance and construct validity among men was later supported by Tylka (2013).

Despite the sound psychometric properties of the BAS, the scale was limited by low factor loadings and differential wording for men and women, which may be burdensome for data collection. Furthermore, the items reflected outdated theoretical foundations for positive body image, which was defined as the absence of negative body image or as inattention to body shape and weight (Tylka and Wood-Barcalow 2015a). Tylka and Wood-Barcalow (2015a) developed Body Appreciation Scale-2. They revised the original BAS items and developed an additional set of items that reflected the newest findings from within the positive body image field. The current version of the BAS-2 consists of 10 items that are answered on a scale from 1 (*Never*) to 5 (*Always*). For a sample of college and MTurk women and men, the scale showed satisfactory factor loadings (λ > 0.62), assumed a unidimensional factor structure (CFI > 0.97, RMSEA < 0.09, SRMR < 0.03), and showed incremental validity in predicting well-being after controlling for other body image constructs (Tylka and Wood-Barcalow 2015a). Apart from the high psychometric qualities of the original scale, follow-up research has supported its internal consistency, unidimensional factor structure, and validity through associations with the related constructs, besides others in Denmark, Sweden, Portugal (Lemoine et al. 2018), Romania (Swami et al. 2017), China (Swami and Ng 2015), Poland (Razmus and Razmus 2017), Spain (Swami et al. 2017), and the Netherlands (Alleva et al. 2016). Overall, the satisfactory psychometric characteristics have been confirmed for various national versions, although not yet in the Czech context.

### Gender and age measurement invariance

In adolescence, gender could influence how body appreciation is formed and experienced. In our society, women and men are targeted by different appearance ideals: women typically by thinness, and men typically by muscularity, although this dichotomy often fades because girls may strive for muscularity as well as boys (Roberts et al. 2022). Furthermore, in the Western socio-cultural context, girls are objectified to a higher extent and socialized to care for their physical appearance and attractiveness (e.g., Daniels et al. 2020). All of these — and the fact that puberty brings adolescent boys closer to the muscular ideal whereas girls move further from the thin ideal (Paquette et al. 2022) — could stand behind the previous research results that found higher body appreciation in adolescent boys than girls (Escoto Ponce de León et al. 2021; Góngora et al. 2020; Paquette et al. 2022).

Investigating gender differences in body appreciation is also warranted in the Czech context. However, to make such a comparison valid, it is critical to establish measurement invariance for girls and boys to confirm that the observed differences can be attributed to the differences in the construct of body appreciation and not to different functions of the scale. Although the need to test measurement invariance has mostly been emphasized for cross-cultural research (e.g., Lacko et al. 2022), examining gender and age-measurement invariance has become standard in validation studies because it ensures that the measurement instruments are valid and reliable across different groups.

The gender invariance has been commonly assessed in BAS-2 research. For example, scalar gender invariance was shown in the original validation study (Tylka and Wood-Barcalow 2015a) for the Polish (Razmus and Razmus 2017), Portuguese (Junqueira et al. 2019; Lemoine et al. 2018), Swedish (Lemoine et al. 2018), and Spanish (Swami et al. 2017) versions. For the French version, only metric gender invariance was examined, and supported (Kertechian and Swami 2017). In Denmark, the scale reached metric and partially scalar invariance due to the non-equivalent intercepts of Item 3 and 8 (Lemoine et al. 2018). However, gender invariance was not successfully established in Romania (Swami et al. 2017). Therefore, it is worth examining whether, in the Czech context and despite the gender-specific experience of body image, body appreciation has the same structure and is measured in the same way by Body Appreciation Scale-2.

Furthermore, adolescence is characterized by substantial physical and psychosocial developmental changes, including those related to body image, such as body shape and weight alterations, the increased awareness of physical appearance, and susceptibility to appearance-related peer pressure (Markey 2010). Accordingly, body appreciation seems to vary during adolescence, when younger adolescents reported higher body appreciation than older adolescents, although with a rather small effect magnitude (Escoto Ponce de León et al. 2021).

While gender invariance has been more thoroughly investigated for the BAS-2, to our best knowledge, age invariance has been insufficiently verified in adolescent samples; only Escoto Ponce de León et al. (2021) examined and supported the scale’s age invariance for Mexican adolescents. Yet, rapid developmental changes in body image and age differences in body appreciation during adolescence urge the need for the comparability of body appreciation across different adolescent age groups.

Overall, these variations call for investigating whether Czech Body Appreciation Scale-2 measures body appreciation in the same manner for girls and boys, and younger and older adolescents, and whether the scores can be used for valid comparisons across these groups.

### Body image research in the Czech context

Similar to other European countries, body image issues are pronounced in the Czech Republic. According to the Health Behavior in School-aged Children survey, 27% of 11-year-old girls and 24% of 11-year-old boys report that they are too fat (Inchley et al. 2020). Among 13-year-olds, it is 32% of girls and 24% of boys, and among 15-year-olds it is 31% of girls and 22% of boys (Inchley et al. 2020). For all these age groups, the proportions of Czech girls and boys who perceive themselves as too fat is close to the European average (11-year-olds: 24% girls, 21% boys; 13-year-olds: 33% girls, 23% boys; 15-year-olds: 36% girls, 22% boys; Inchley et al. 2020). Also, in the Czech Republic, only 55% of 18-year-old girls and 44% of 18-year-old boys report being satisfied with their weight, 50% of girls disclose wanting to lose weight, and 29% to put on weight (Šmídová et al. 2018). However, there is a lack of evidence for positive body image among adolescents in the Czech Republic. To our knowledge, only one study focused on body appreciation in the Czech Republic. It found that exposure to body positivity online was only marginally associated with higher body satisfaction through higher body appreciation among Czech adolescents aged 13 to 18 (Kvardova et al. 2022). The need to measure the body appreciation of Czech adolescents is critical, particularly given the high prevalence of body dissatisfaction (Inchley et al. 2020; Šmídová et al. 2018). We believe that this validation study of the BAS-2 could encourage this line of research in the Czech context.

## The current study

The validity of Body Appreciation Scale-2 (Tylka and Wood-Barcalow 2015a) has been evidenced for the original scale and its many translated versions (Alleva et al. 2016; Behrend and Warschburger 2022; Casale et al., 2021; Lee 2023; Lemoine et al. 2018; Ma et al. 2022; Razmus and Razmus 2017; Swami et al. 2017; Swami et al. 2017), but not yet in the Czech context. The present study examined the internal consistency and factor structure of Body Appreciation Scale-2 (Tylka and Wood-Barcalow 2015a), as adapted to the Czech language in an adolescent sample. Because of gender and age variations in body appreciation (Escoto Ponce de León et al. 2021), we further investigated the measurement invariance among girls and boys, and younger (13–15) and older (16–18) adolescents. Furthermore, the validity of Czech Body Appreciation Scale-2 (Tylka and Wood-Barcalow 2015a) was examined through associations with body satisfaction; media-ideal internalization, which in this study refers to adopting attractive thin (for girls) and muscular (for boys) body appearance from social media; appearance schematicity, which involves heightened attention to appearance-related stimuli, investment in physical appearance, and the belief that appearance has a significant impact on one’s life (Hargreaves and Tiggemann 2002); self-esteem; and depression. Based on the relationships found in prior literature (e.g., Alleva et al. 2016; Lemoine et al. 2018; Razmus and Razmus 2017; Swami et al. 2017; Swami et al. 2017), we expected body appreciation to be positively correlated with body satisfaction and self-esteem, and negatively correlated with media-ideal internalization, appearance schematicity, and depression. We expected body satisfaction to be strongly associated with body appreciation (Tylka and Wood-Barcalow 2015a), but not enough to indicate that they are indistinguishable and unique concepts (i.e., they show sufficient discriminant validity). Lastly, because of the scarce knowledge of gender differences in body appreciation in adolescence, we explored the gender and age differences in body appreciation, and the gender differences in the relationship of body appreciation with the above-mentioned body image and well-being constructs.

## Methods

### Participants and procedure

The present study used two samples. Sample 1 was used for the BAS-2’s descriptive statistics, internal consistency, factor structure, measurement invariance, and associations with media-ideal internalization, appearance schematicity, self-esteem, and depression. Sample 2 was used to examine the BAS-2’s factor structure and the association between body appreciation (BAS-2) and body satisfaction. Samples 1 and 2 were collected independently of each other; Sample 1 in August 2021 and Sample 2 in November 2020.

Data were collected by Median, s.r.o., a survey agency, through an online survey. The Computer-Assisted Web Interviewing (CAWI) method was used to distribute online questionnaires via website links and save the data in an electronic form. The majority of the Median panel was recruited offline. The participants were sampled from the agency’s online panel. Prior to participation, the agency obtained informed consent from the adolescent and one parent. Refusal to participate could be announced at any time before and during the study. Adolescents filled out the questionnaires at home. The anonymity of participation and the responses was guaranteed. The option to answer “I don’t know or prefer not to say” was provided. Data for this study were collected within the project Modeling the future: Understanding the impact of technology on adolescent’s well-being (FUTURE). The data collection was approved by the Research Ethics Committee of Masaryk University (ref. number: EKV-2018-068). Since this study used data that were already collected, we did not apply power analysis or other rules to determine its sample size.

#### Sample 1

Sample 1 consisted of 613 Czech adolescents (52% girls) aged 13–18 (*M* = 15.5, *SD* = 1.7). This sample was collected as part of a larger survey of 1751 adolescents with quota sampling to represent Czech households with children according to household income and region (NUTS2). Within the sample, participants were randomly assigned to one of three studies, including the current one, with quotas for a balanced representation of gender and age for each study. The agency excluded participants who did not respond to their call, were out of the target group, completed the survey in an excessively short time, or had more than 10% missing data. The questionnaire took on average 17 min, and participants received a reward of 2–3 euros. Since the main purpose of this data collection was an experimental study of the social media comments, the study was introduced as “research on attention and memory in relation to Instagram images”. The scales of depression, media-ideal internalization, body appreciation, self-esteem, and appearance schematicity were, in this order, administered to participants in the first part of the study, prior to the experimental stimuli exposure.

#### Sample 2

Sample 2 consisted of 1,530 Czech adolescents (50% girls) aged 13–18 (*M* = 15.4, *SD* = 1.7). Quota sampling was used, with representative distributions for gender, age, household income, region (NUTS3), and municipality size. The final sample was selected from an initial pool of 12,664 adolescents. Those who were excluded from the study did not respond, did not meet the eligibility criteria, or had more than 10% missing data. The questionnaire, which besides the body appreciation and body satisfaction scales, included scales for social (e.g., social support from family and friends), physical (e.g., perceived health status), and psychological (e.g., happiness) well-being and online experiences (e.g., meeting unknown people from the internet), took an average of 25 min, and participants received a remuneration of 4 euros. Since the whole project focused on the media use of adolescents and the associated factors, the study was advertised as “a study of online experiences and the associated factors among youth”. Body satisfaction and body appreciation were measured, in this order, after the basic questions about gender, age, frequency of internet and Instagram use, and the frequency of viewing body positivity online.

### Translation and cognitive interviews

The scales were translated with a modified collaborative iterative translation process that involved multiple experts (Douglas and Craig 2007), together with cognitive interviews (e.g., Wildy and Clarke 2009) that were used instead of the pilot testing, as described by Douglas and Craig (2007). Firstly, all scales were translated by the authors, one of which has a M.A. in Psychology and the other who is an Associated Professor of Psychology. Given that all of the experts already had extensive experience in conducting similar surveys in the Czech Republic and the fact that they are familiar with relevant linguistic equivalents, parallel translation was not necessary in this phase (Douglas and Craig 2007). Secondly, a consultation about the initial translation was iteratively made for the accuracy and suitability with one other expert researcher, who is a doctoral student in Psychology. By consensus, some items were slightly amended to the most suitable translation. The translation that best corresponded to the original wording in terms of the content and meaning was used. Item 8 of the BAS-2 (i.e., “My behavior reveals my positive attitude toward my body; for example, I hold my head high and smile”) was, by the decision of the first author and following a discussion with other team members, slightly changed; the example (i.e., “for example, I hold my head high and smile”) was omitted because it was not meaningful in the Czech language and context. Thirdly, prior to the data collection, the comprehension of all of the used items was tested via cognitive interviews with five adolescents aged 15 and 16 (three boys, two girls). No significant modifications were required, and only minor adjustments were made during the interviews to ensure the age-appropriateness of a few terms in the translations. The Czech version of the BAS-2 can be found in the Additional file 1.

### Measures

The measures of body appreciation, internalization of thin/muscular ideal, appearance schematicity, self-esteem, and depression were used in the data collection for Sample (1) Body appreciation and body satisfaction were measured with Sample (2) Since all of the measures were not previously adapted in the Czech context, we translated them for the purpose of this and other studies conducted within the FUTURE project. The Czech translations that are not protected by copyright are attached in the Additional file 1. To document the scales’ reliability and validity, we additionally analyzed the data from 2,500 Czech adolescents aged 11–16 (*M* = 13.4, *SD* = 1.7) (50% girls), which was collected within the FUTURE project in June 2021. We refer to this as Sample (3) These data were collected through an online survey by STEM/MARK, a. s., and Data Collect, s. r. o. The agencies recruited participants based on equal distributions of gender and age, and with the socioeconomic status and place of residence for Czech households with children. The questionnaire focused on ICT usage (e.g., social media, mHealth apps) and well-being (e.g., physical activity, peer support, loneliness) in Czech adolescents. Adolescents could always answer “I don’t want to respond” and they were debriefed about the study purpose after completing the questionnaire. The median time of completion was 22 min. From this data, we report reliability and validity evidence for the media-ideal internalization, self-esteem, and depression scales below.

#### Body appreciation

Body appreciation was measured with Body Appreciation Scale-2 (Tylka and Wood-Barcalow 2015a). The 10 items were answered on a scale that ranged from 1 (Never) to 5 (Always). The higher score indicated higher body appreciation. The following instruction was provided: “Now, please read the following statements and indicate how often this happens to you.“

#### Body satisfaction

Body satisfaction was measured with five items of Body Dissatisfaction Subscale of the Eating Disorder Inventory-3 (Garner 2003). The original scale consists of both positively phrased and negatively phrased items; in this study, we only used the positively phrased ones that captured satisfaction with one’s thighs, abdomen, hips, buttocks, and overall body shape. The five items that were omitted inquired about dissatisfaction with thighs, abdomen, hips, and buttocks, and the last retained item detected eating disorder symptoms. The items were answered on a scale that ranged from 1 (Very untrue of me) to 5 (Very true of me). The original validation study of EDI-3 showed satisfactory reliability and validity for the Body Dissatisfaction Subscale on a clinical sample of adolescents: high internal consistency (α = 0.93), high test-retest reliability (*r* = .95), high overall factor loadings (> 0.45), and the expected associations with other body image constructs (Garner 2003). Similarly, our data showed satisfactory internal consistency (ω = 0.797) and the CFA results supported the original unidimensional factor structure: *X*^2^(5) = 143.187, CFI = 0.928, RMSEA = 0.157 [90% CI: 0.135, 0.179], SRMR = 0.056, with the exception of RMSEA, which was higher than the recommended threshold (0.080). However, the RMSEA may not be a reliable indicator of model misspecification for models with small degrees of freedom (Kenny et al. 2015). Since our model had five df, we considered the model fit sufficient.

#### Media-ideal internalization

Media-ideal internalization was measured with the Sociocultural Internalization of Appearance Questionnaire – Adolescents (SIAQ-A; Keery et al. 2004). The five items were answered on a scale that ranged from 1 (Definitely disagree) to 5 (Definitely agree). The items (e.g., “I want my body to look like theirs”) were adapted from an assessment of internalization from the traditional media to social media. We provided instructions to answer in regards to social media (i.e., “On social media, you can also sometimes see photos or videos of girls you think have a great body [version for girls] / boys you think have a great body [version for boys]. When you see girls/boys like that on social media, how much does the following apply to you…”). The original version of the scale showed satisfactory internal consistency (α = 0.83–0.92) and the expected correlations for body image constructs, providing support for its validity (Keery et al. 2004). Similarly, the current data showed satisfactory internal consistency (ω = 0.862) and the CFA results supported the unidimensional factor structure: *X*^2^(4) = 12.266, CFI = 0.993, RMSEA = 0.073 [90% CI: 0.025, 0.121], SRMR = 0.020. To provide additional evidence for SIAQ-A’s psychometric qualities, on Sample 3, the scale exhibited good internal consistency (ω = 0.922) and high factor loadings (> 0.81), although some fit indexes were lower than ideal, probably because of the high residual correlation between two similarly-worded items: “I want my body to look like theirs” and “I would like to look just like them” (*r* = .144); CFI = 0.903, TLI = 0.805, RMSEA = 0.276 (90%CI: 0.261; 290), SRMR = 0.044.

#### Appearance schematicity

Appearance schematicity was measured with 10 items of the Appearance Schemas Inventory-Revised (ASI-R; Cash et al. 2004). Two items of the original scale were omitted: one that was a duplicate and reversely asked about the impact of perceived attractiveness on emotional well-being, and one that focused on the impact of controlling physical appearance on social and emotional events that was hard to translate into the Czech language. The items were answered on a scale that ranged from 1 (Definitely disagree) to 5 (Definitely agree). In the validation study of Cash et al. (2004), the ASI-R Self-Evaluative Salience Subscale had satisfactory internal consistency (α = 0.82), quite high loadings extracted within the Principal Component Analysis (0.49–0.74), and it correlated significantly with other body image constructs (e.g., internalization of societal ideals), supporting the ASI-R’s validity. Similarly, our data showed satisfactory internal consistency (ω = 0.897) and CFA results that supported the original unidimensional factor structure: *X*^2^(26) = 80.152, CFI = 0.971, RMSEA = 0.070 [90% CI: 0.053, 0.088], SRMR = 0.028.

#### Self-esteem

Self-esteem was measured with five items of the Rosenberg Self-Esteem Scale (RSES; Rosenberg 2015). The omitted negatively phrased items were “At times I think I am no good at all”, “I feel I do not have much to be proud of”, “I certainly feel useless at times”, “I wish I could have more respect for myself”, and “All in all, I am inclined to feel that I am a failure”. The items (e.g., “I feel that I have a number of good qualities”) were answered on a scale that ranged from 1 (Completely untrue) to 4 (Completely true). The higher score indicated higher self-esteem. In the study of Sinclair et al. (2010), the RSES showed high internal consistency (α = 0.91), high component-item correlations (*r* = .66–0.85), which were extracted within the Principal Component Analysis, and presumed correlations with well-being concepts (e.g., depression), which supported the scale’s validity. The current data also showed satisfactory internal consistency (ω = 0.879), and CFA supported the original unidimensional factor structure: *X*^2^(5) = 29.750, CFI = 0.976, RMSEA = 0.111 [90% CI: 0.074, 0.152], SRMR = 0.027, with the exception of RMSEA. However, since the model had only five degrees of freedom, we considered it satisfactory. Finally, Sample 3 also featured the shortened RSES, which consisted of positively-phrased questions. Internal consistency was good (ω = 0.89) and factor loadings were above 0.75. The model fit estimated on this data was less than ideal, probably because of the residual correlation between the first two items of RSES (i.e., “On the whole, I am satisfied with myself”, “I take a positive attitude toward myself”) or the few degrees of freedom (5); CFI = 0.912, TLI = 0.824, RMSEA = 0.224 (90%CI: 0.209; 0.238), SRMR = 0.046.

#### Depression

Depression was measured with four items of the scale of depressive moods developed by Kandel and Davies (1982). The items captured how often adolescents experienced depressive moods in the last months (e.g., “I felt unhappy or sad”). Two unused items were “Feeling too tired to do things” and “Having trouble going to sleep or staying asleep”. The items were answered on a scale that ranged from 1 (Never) to 5 (Very often). To demonstrate the scale’s reliability and validity, Kandel and Davies (1982) reported sufficient internal consistency (α = 0.79), test-retest reliability (*r* = .76), and correlations with other depression indicators, which documented the validity of the scale. Our current data showed satisfactory internal consistency (ω = 0.866), sufficiently high factor loadings (> 0.73), and a unidimensional factor structure; *X*^2^(2) = 0.992, CFI = 1.000, RMSEA < 0.000 [90% CI: 0.000, 0.079], SRMR = 0.005. Additionally, on Sample 3, the scale’s internal consistency was satisfactory (ω = 0.856). Factor loadings were high (> 0.74) and the one-factor model fit the data well; CFI = 0.991, TLI = 0.974, SRMR = 0.014, RMSEA = 0.086 (90%CI: 0.063; 0.110).

### Data analysis

Data analysis was performed in R (v4.1.2; R Core Team 2021), and the packages lavaan (Rosseel 2012), semTools (Jorgensen et al. 2021), and MVN (Korkmaz et al. 2014). All of the variables, except for gender and age, were modelled as latent within the confirmatory factor analysis (CFA) and the Structural Equation Modelling (SEM). On Sample 1, we computed the BAS-2’s descriptive statistics, internal consistency, factor structure, and measurement invariance, and associations with media-ideal internalization, appearance schematicity, self-esteem, and depression. The factor structure was examined with CFA. To fix the scale in the CFA models, the factor loading of the first indicator was fixed to 1 and others were freely estimated. The measurement invariance was evaluated with the multigroup confirmatory factor analyses (MG-CFA) for (a) girls and boys and (b) younger (13–15) and older (16–18) adolescents. We compared the model fit indices (CFI, TLI, RMSEA, SRMR) for increasingly restricted nested models, namely the configural (i.e., fixed factor structure), metric (i.e., fixed loadings), and scalar (i.e., fixed intercepts) models. When evaluating the model fit, we followed the criteria for good/acceptable model fit for the configural model proposed by Hu and Bentler (1999): TLI ≥ 0.95/0.90, CFI ≥ 0.95/0.90, RMSEA ≤ 0.06/0.08, SRMR ≤ 0.08, and the criteria for metric and scalar invariance proposed by Chen (2007): ΔCFI ≤ 0.01, ΔRMSEA ≤ 0.015, ΔSRMR ≤ 0.030 (metric level)/ 0.015 (scalar level). When comparing the fit indices for the levels of measurement invariance, we used delta (Δ) to indicate model fit change. To test the differences between the groups (i.e., girls versus boys, younger versus older adolescents), latent mean comparison (i.e., structured means modeling, Sörbom 1974) was applied. We used the reference group method (i.e., setting the latent mean in one group to 0 while allowing it to vary in the remainder of the groups). The associations with other constructs were assessed by investigating latent correlations via SEM. Sample 2 was used to test the BAS-2’s factor structure and the association between body appreciation and body satisfaction. This association was assessed with the heterotrait-monotrait ratio of correlations (HTMT). To assess whether boys and girls differed in the strength of the associations between the BAS-2 and the other related constructs, in both Sample 1 and 2, we used multigroup structural equation modeling (MG-SEM) with established metric invariance between boys and girls. The difference was verified by comparing two nested models with a chi-square difference test, one with a covariance constrained to be equal across genders and the second with an unconstrained covariance. This procedure was repeated for each latent correlation. We also evaluated the factor structure of other used measures with the CFA, and reported the results in the Measures part of the Methods section.

### Transparency and openness

All work (e.g., papers, packages) that we used is appropriately cited in the text and in the References section. The data that support the current findings are freely available at: osf.io/ybkvn. The R code used for the analyses is included in the Additional file 2. The Czech version of the main scale (i.e., BAS-2) and the other scales translated into Czech are attached in the Additional file 1. The other scales are in their original wording available in the papers cited in the Measures section, except for ASI-R (Cash et al. 2004) and EDI-3 (Garner 2003), which are under paid copyright. This study was not preregistered. We report our sample size rationale, all data exclusions (if any), all manipulations, and all measures in the study.

## Results

### Data analysis assumptions

The data showed multivariate non-normality, as indicated by the Henze-Zirkler test (*HZ* = 3.598, *p* < .001). All items also showed univariate non-normality; all Shapiro-Wilk tests were statistically significant (*p* < .001). With five and more categories (Rhemtulla et al. 2012) and such high nonnormality (Li 2016), it is possible to analyze data with a robust continuous estimator. Therefore, a Robust Maximum Likelihood (MLR) estimator was used in the analyses. We used Full Information Maximum Likelihood (FIML) to handle missing values.

#### Descriptive statistics and internal consistency

Descriptive statistics and internal consistency were computed on Sample 1. Table 1 displays means, standard deviations, factor loadings, and item-total correlations for the total sample, and for girls and boys, separately. Item-rest correlations were acceptably high (> 0.64 for girls, > 0.67 for boys). The internal consistency indicated by the McDonald’s omega was satisfactory, ω = 0.939. The average extracted variance was adequate as well, AVE = 0.610.

**Table 1:** Means, standard deviations, factor loadings, and item-total correlations from Sample 1

Item	Girls			Boys			Total sample		
	M (SD)	λ	ITC	M (SD)	λ	ITC	M (SD)	λ	ITC
1. I respect my body	3.7 (1.1)	0.82	0.85	3.8 (1.1)	0.84	0.85	3.7 (1.1)	0.82	0.80
2. I feel good about my body	3.5 (1.1)	0.87	0.86	3.7 (1.1)	0.84	0.84	3.6 (1.1)	0.85	0.81
3. I feel that my body has at least some good qualities	3.9 (1.0)	0.74	0.78	3.9 (1.0)	0.78	0.81	3.9 (1.0)	0.75	0.74
4. I take a positive attitude towards my body	3.6 (1.1)	0.90	0.90	3.7 (1.0)	0.85	0.85	3.7 (1.1)	0.88	0.84
5. I am attentive to my body’s needs	3.5 (1.0)	0.55	0.64	3.5 (1.0)	0.60	0.67	3.5 (1.0)	0.56	0.58
6. I feel love for my body	3.,6 (1.1)	0.88	0.89	3.8 (1.1)	0.87	0.87	3.7 (1.1)	0.87	0.84
7. I appreciate the different and unique characteristics of my body	3.5 (1.1)	0.74	0.79	3.6 (1.1)	0.75	0.80	3.5 (1.1)	0.74	0.74
8. My behavior reveals my positive attitude toward my body	3.3 (1.1)	0.72	0.76	3.5 (1.1)	0.75	0.79	3.4 (1.1)	0.73	0.72
9. I am comfortable in my body	3.7 (1.1)	0.83	0.83	3.9 (1.0)	0.86	0.86	3.8 (1.0)	0.84	0.80
10. I feel like I am beautiful even if I am different from media images of attractive people (e.g., models, actresses/actors)	3.4 (1.1)	0.73	0.78	3.4 (1.2)	0.70	0.75	3.4 (1.1)	0.71	0.70

### Factor structure

On Sample 1, the model showed a good fit to the data, *X*^2^(35) = 88.463, *p* < .001, CFI = 0.982, TLI = 0.977, RMSEA = 0.060 [90% CI: 0.045, 0.076], SRMR = 0.024. Despite the statistical significance of the chi-square test, we did not regard this result as an indication of poor model fit because of the large sample size. All fit indices met the criteria for model fit by Hu and Bentler (1999).

Table 1 shows factor loadings for the whole sample, and for girls and boys, separately. The factor loadings were acceptably high among the total sample (λ > 0.56), as well as among the girls (λ > 0.55) and boys (λ > 0.58) subsamples. Item 5 (i.e., “I am attentive to my body’s needs”) had a lower factor loading than the others (λ = 0.56; λ = 0.55 among girls, λ = 0.58 among boys), but it was still acceptable.

Additionally, we replicated the factor structure of the BAS-2 scale on Sample 2 (*N* = 1,530). This model exhibited a good fit as well, *X*^2^(35) = 267.149, *p* < .001, CFI = 0.963, TLI = 0.952, RMSEA = 0.080 [90% CI: 0.072, 0.090], SRMR = 0.029, with high factor loadings (λ > 0.52; 8 items > 0.73), supporting the presumed factor model of the BAS-2. Similar to the first model, the fit indices met the standard criteria according to Hu and Bentler (1999).

### Measurement invariance

#### Gender

To discover if Body Appreciation Scale-2 measures body appreciation equivalently for girls and boys, we tested the measurement invariance for gender, using Sample 1. First, we computed the configural model that examines the equivalence of the factor structure and then proceeded to the metric (i.e., factor loadings equivalence) and scalar (i.e., intercepts equivalence) models. The configural model fit the data reasonably, CFI = 0.980, TLI = 0.974, RMSEA = 0.064 [90% CI: 0.048, 0.081], SRMR = 0.028. The fit of the metric model did not show substantial differences from the configural model, ∆CFI < 0.001, ∆TLI = − 0.003, ∆RMSEA = − 0.003, ∆SRMR = 0.010. The attained metric invariance suggests that the BAS-2 factor variance and covariance are comparable among adolescent girls and boys (Lacko et al. 2022). Similarly, the scalar model did not show substantial difference in the model fit from the metric model, ∆CFI = 0.003, ∆TLI < 0.001, ∆RMSEA = < 0.001, ∆SRMR = 0.003. Therefore, scalar invariance was supported, meaning that the latent means in body appreciation, measured by Body Appreciation Scale-2, can be compared.

#### Age

Besides gender equivalence, we also looked at the measurement invariance for age, using Sample 1 as well. We created two age categories (i.e., 13–15, 16–18) and investigated measurement invariance across them. Identical to the case of gender invariance, we gradually examined the configural, metric, and scalar models. The configural model showed a good fit to the data, CFI = 0.978, TLI = 0.972, RMSEA = 0.067 [90% CI: 0.050, 0.083], SRMR = 0.029. The metric model did not significantly differ from the scalar model, ∆CFI < 0.001, ∆TLI = − 0.003, ∆RMSEA = − 0.004, ∆SRMR = 0.012. This result showed that factor variance and covariance can be compared among the respective age groups. Moreover, the scalar model did not substantially differ from the metric model in the model fit, ∆CFI = 0.001, ∆TLI = − 0.002, ∆RMSEA = − 0.002, ∆SRMR = 0.001. The data thus supported scalar invariance across the age groups; latent means can hence be compared.

### Comparison of latent means

On Sample 1, we also examined gender and age differences in the BAS-2 scores. Girls showed a lower estimated latent mean in the BAS-2 (*M* = − 0.076, *SE* = 0.050, *SD* = 0.913) than boys (*M* = 0.055, *SE* = 0.051, *SD* = 0.884). This difference was, however, statistically insignificant, ∆χ^2^ = 3.036, ∆df = 1, *p* = .081. The latent mean difference (*M* = − 0.131 [95% CI: − 0.277, 0.016], *SE* = 0.075), as well as its effects size (*d* = − 0.146), were rather small. As for age, younger participants showed a smaller estimated latent mean (*M* = − 0.028, *SE* = 0.052, *SD* = 0.920) than their older counterparts (*M* = 0.034, *SE* = 0.049, *SD* = 0.883). However, a chi-squared difference test between the nested models showed that these differences are insignificant, ∆χ^2^ = 0.695, ∆df = 1, *p* = .404. The latent mean difference was only − 0.062 (CI: − 0.209, 0.085; *SE*: 0.075) and Cohen’s *d* was negligible, *d* = − 0.069.

### Associations with other constructs

To examine the validity of the BAS-2, this study also looked into its associations with media-ideal internalization, appearance schematicity, self-esteem, depression, and body satisfaction. The connections with media-ideal internalization, appearance schematicity, self-esteem, and depression were studied on Sample 1 (*N* = 613, aged 13–18, 52% girls). The model fit the data adequately, *X*^2^(483) = 919.406, *p* < .001, CFI = 0.960, TLI = 0.956, RMSEA = 0.042 [90% CI: 0.038, 0.046], SRMR = 0.046. Body appreciation measured by the BAS-2 correlated negatively with media-ideal internalization, *r* = − .477 [95% CI: − 0.556, − 0.399], *p* < .001, appearance schematicity, *r* = − .393 [95% CI: − 0.480, − 0.306], *p* < .001, and depression, *r* = − .397 [95% CI: − 0.487, − 0.308], *p* < .001. Contrarily, positive correlation appeared between body appreciation (BAS-2) and self-esteem, *r* = .841 [95% CI: 0.803, 0.878], *p* < .001.

The gender differences in the strengths of the above-mentioned associations were, on Sample 1, verified with MG-SEM. The configural model fit the data well, *X*^2^(966) = 1470.928, *p* < .001, CFI = 0.954, TLI = 0.950, RMSEA = 0.044 [90% CI: 0.040, 0.049], SRMR = 0.052, and metric invariance for the whole model was also established, ∆CFI = 0.001, ∆TLI < 0.001, ΔRMSEA < 0.001, ΔSRMR = 0.011. Furthermore, we tested invariance for the individual scales; the full results can be found in the Additional file 1. The metric invariance was also mostly supported for the individual scales. The change in the model fit slightly exceeded the used criteria (Hu and Bentler 1999; Chen 2007) for the depression and body satisfaction scales, and the fit of the configural model was slightly worse than the cut-off criteria for body satisfaction. Yet, such results may not be surprising with respect to the smaller number of indicators for both scales. Given the reached metric invariance of the whole model as we report above, we believe the gender differences can be interpreted validly. Chi-squared difference tests showed statistically significant differences in the relationships of the BAS-2 with media-ideal internalization (Δ*X*^2^ = 9.2251, Δdf = 1, *p* = .002) and depression (Δ*X*^2^ = 5.4346, Δdf = 1, *p* = .020) between boys and girls. In both associations, girls showed stronger correlations than boys (*r* = − .586 versus − 0.337 and *r* = − .480 versus − 0.273, respectively). The other associations were not moderated by gender (appearance schematicity Δ*X*^2^ = 0.8367, Δdf = 1, *p* = .360; self-esteem Δ*X*^2^ = 0.26215, Δdf = 1, *p* = .609). The association with body satisfaction was studied on Sample 2 (*N* = 1,530, aged 13–18, 50% girls). First, the latent correlation was assessed. The model that examined the association between body appreciation (BAS-2) and body satisfaction showed a good fit to the data, *X*^2^(89) = 616.649, *p* < .001, CFI = 0.947, TLI = 0.938, RMSEA = 0.070 [90% CI: 0.064, 0.075], SRMR = 0.040. Body appreciation correlated positively with body satisfaction, *r* = .745 [95% CI: 0.709, 0.781], *p* < .001. For the purposes of the moderation effect of gender, a metric invariance was established (ΔCFI = 0.005, ΔTLI = 0.002, ΔRMSEA < 0.001, ΔSRMR = 0.011). We found that girls showed a stronger correlation than boys according to a chi-squared difference test, Δ*X*^2^ = 23.709, Δdf = 1, *p* < .001 (*r* = .786 versus 0.676). Even though these two constructs are closely related, the HTMT showed sufficient evidence for discriminant validity (HTMT = 0.701), suggesting that both constructs are unique, consistent with our presumptions.

## Discussion

The present study examined the validity of the Czech version of Body Appreciation Scale-2 (Tylka and Wood-Barcalow 2015a) among adolescent girls and boys in the Czech Republic. Body Appreciation Scale-2 measures body appreciation, a construct that has frequently been studied within the positive body image field (e.g., Andrew et al. 2015; Homan and Tylka 2018; Tiggemann and McCourt 2013). The original scale has satisfactory psychometric characteristics (Tylka and Wood-Barcalow 2015a), yet the data on its validity in adolescence, a sensitive developmental phase for body image (Markey 2010), in the Czech context, were lacking. To the best of our knowledge, the BAS-2’s age invariance has also not been sufficiently examined among adolescents; only one recent study (Escoto Ponce de León et al., 2021) targeted this issue. Utilizing two samples of Czech adolescents, aged 13–18, which had balanced age and gender proportions (i.e., girls, boys), the current study demonstrated satisfactory internal consistency, the expected factor structure, and gender and age invariance. It also confirmed the BAS-2’s validity via its associations with the related body image and well-being constructs (Tylka and Wood-Barcalow 2015a). The present study documented that the Czech version of the scale can be utilized to assess body appreciation among Czech adolescents, including the comparison of the BAS-2 scores between adolescent girls and boys and the respective age groups.

### Items characteristics, factor structure, and internal consistency

The internal consistency and the average variance extracted for the BAS-2 were adequately high. The item-total correlations were also adequate among both girls and boys. Similarly, factor loadings were sufficiently high among the total sample, and for girls and boys separately. The unidimensional factor model had a satisfactory fit to the data; the fit indices met the criteria suggested by Hu and Bentler (1999). We additionally replicated the unidimensional factor structure on the second sample of adolescents, which brought further evidence for the validity of the Czech version of the BAS-2. Our results align with the prior studies that have documented satisfactory internal consistency and a good data model fit among adolescents (Escoto Ponce de León et al. 2021; Góngora et al. 2020; Lemoine et al. 2018; Paquette et al. 2022).

Although all factor loadings were acceptable, it is noteworthy that Item 5 (i.e., I am attentive to my body’s needs) had a slightly lower loading. This lower factor loading also appeared in the previous studies (e.g., Alleva et al. 2016; Kertechian and Swami 2017; Lemoine et al. 2018; Swami et al. 2017b). One reason may be that “body’s needs” may be vague, and it can be difficult to recall the specific needs of the body. Another cause may stem from the different construct for the item. For instance, the Body Investment Scale (Orbach and Mikulincer 1998) measures the concept of body care through more specific items, such as “I like to pamper my body” or “I use body care products regularly”. On the other hand, caring for the body’s needs poses a significant component of the body appreciation construct (e.g., Wood-Barcalow et al. 2010). It must be noted that, despite being lower, the loading of Item 5 was still in an acceptable range. Yet, it would be helpful to focus on this issue in future research. The follow-up studies could, for instance, refine the item by providing a specific example of a body’s needs and compare the results.

### Measurement invariance: gender and age

Our results showed the gender invariance of the BAS-2 at the scalar level, meaning that factor structure, factor loadings, and intercepts were equivalent for both girls and boys. This is consistent with prior evidence that supported the BAS-2’s gender invariance in adolescent (Escoto Ponce de León et al. 2021; Lemoine et al. 2018) and adult samples (Junqueira et al. 2019; Kertechian and Swami 2017; Swami et al. 2017; Tylka and Wood-Barcalow 2015a). However, it should be noted that our results for gender differences in body appreciation contrasted with previous research (Escoto Ponce de León et al., 2021; Góngora et al. 2020; Paquette et al. 2022). The above-mentioned studies found that adolescent girls had significantly lower body appreciation than boys, possibly because of the higher unattainability of societal ideals for girls, as Paquette et al. (2022) suggested, or because of higher objectification (Daniels et al. 2020). While this pattern occurred in our study as well, the difference was small and statistically insignificant. Such a discrepancy could lie in the different approaches to testing the gender differences in body appreciation: Whereas the above-mentioned research tested the difference in observed scores, we examined the latent scores. Our results suggest that, on average, Czech adolescent girls and boys appreciate their bodies to a similar extent. Yet, future research should dig deeper into why gender differences in body appreciation could be mitigated in the Czech context, or if this finding can be generalized for adolescents from other countries as well.

We also examined age invariance. The results showed that the BAS-2 functioned equivalently across the 13–15 and 16–18 age groups, and the scalar age invariance was supported. This is consistent with the results of Escoto Ponce de León et al. (2021), which also supported the BAS-2’s age invariance among adolescents. As in the case of gender differences in body appreciation, older adolescents (16–18) reported higher body appreciation than the younger group (13–15), yet this difference was not statistically significant. Although age differences in adolescent body appreciation would be expected given the rapid body image changes (e.g., Markey 2010), our findings are consistent with Escoto Ponce de León et al. (2021), who also reported negligible age differences in the body appreciation of adolescents, yet contrasts with the research on adult body appreciation, which found that body appreciation significantly increases with age (Tiggemann and McCourt 2013). It is possible that, despite adolescence being a critical phase for body image development (Markey 2010), significant age differences in body appreciation do not appear in this relatively short period, as opposed to the long span of adulthood. Overall, despite these nuances, our findings indicate that the BAS-2 can be used to examine gender and age differences in body appreciation among adolescents.

### Associations with other constructs

To examine the validity of the BAS-2, we also looked into the relationships with other constructs. Overall, the results showed patterns of associations that corresponded to the expectations based on theory and prior research. Body appreciation correlated moderately and negatively with thin- (girls) and muscular- (boys) ideal internalization. This supported the notion that adolescents who are more appreciative of their bodies internalize media ideals to a lesser extent (Tylka and Wood-Barcalow 2015a). A moderate negative correlation also appeared with depression, corroborating the previous results on depression (Winter et al. 2019) and affect in general (Razmus and Razmus 2017; Swami et al. 2017b). Despite the limited evidence on the relationship between body appreciation and appearance schematicity, we expected them to be negatively correlated. Appearance-schematic individuals consider physical appearance central to their worth and view attractiveness as significant (Hargreaves and Tiggemann 2002), while body appreciation involves cherishing the body regardless of its physical appearance, accepting it with all its attributes (even the ones that do not fit the societal notion of beauty), and resisting unrealistic appearance ideals (Tylka 2013). The current findings supported our presumption and showed a medium-strong negative correlation between body appreciation and appearance schematicity. The present study also demonstrated a positive correlation with self-esteem, which is consistent with the prior research (Alleva et al. 2016; Lemoine et al. 2018; Razmus and Razmus 2017; Tylka and Wood-Barcalow 2015a). It should be noted that the association (*r* = .841 [95% CI: 0.803, 0.878]) was slightly higher than in the above-referred studies (*r* = .50–0.71). Perhaps, body appreciation may be more crucial to the overall self-evaluation of adolescents (i.e., the present sample) than the adult population (i.e., the previous samples), which is consistent with the notion of the increased importance of body image in adolescence (Markey 2010). Also, the previous studies reported the means of observed variables, whereas the current study reported latent means, which may have caused the difference. Lastly, we studied the relationship between body appreciation and body satisfaction. We expected that body appreciation and body satisfaction would be closely associated, yet not entirely overlap (e.g., Tylka and Wood-Barcalow 2015a). Consistent with the previous data (e.g., Swami et al. 2017; Tylka and Wood-Barcalow 2015a), body appreciation was strongly positively correlated with body satisfaction (*r* = .745 [0.709, 0.781]), which supports the validity of the Czech BAS-2.

In the exploratory part, we discovered several gender differences in the association between body appreciation and the constructs mentioned above. They do not have evident implications for the scale’s validity, given the lack of a theory about such differences. Yet, they bring needed evidence to the gender and age patterns in the connections between body appreciation and other body image and well-being characteristics in adolescence, and they may be relevant to future research in this area. However, caution is necessary when interpreting these findings: first, because of their purely exploratory nature and, second, because the scales were not previously validated in the Czech context, which poses a threat to the validity. While the association of body appreciation with self-esteem and appearance schematicity did not differ by gender, we discovered that body appreciation was more closely connected with body satisfaction, media-ideal internalization, and depression among girls than boys. This is a novel finding that indicates that the presumed protective role of body appreciation (Wood-Barcalow et al. 2010) may be more accentuated for adolescent girls. Yet, the reciprocal effect, especially between body appreciation and media-ideal internalization, which would be consistent with the Tripartite Influence Model of Body Image (Thompson et al. 1999), is also plausible; internalization decreases body appreciation, which may be accentuated for girls, as our results showed. The associations between body appreciation, body satisfaction, and depression may be reciprocal as well, and the cross-sectional data, unfortunately, did not enable us to disentangle them. Future research needs to shed more light on gender differences in the role of body appreciation on well-being. And, in light of our study’s limitations, it would be fruitful to replicate these results after providing more evidence for the validity of the utilized scales in the Czech context.

### Limitations and future research

The present study has several limitations. The body satisfaction, self-esteem, and depression scales were not administered at their full length. Since the items omitted from the measurement of body satisfaction reversely inquired about satisfaction with the same body parts, we believe that the content validity was maintained. Yet, we do recognize the insufficient evidence of the shortened version’s validity as a drawback. Further, the negatively-phrased self-esteem items were left out because of the limited space and their malfunction in previous research (e.g., DiStefano and Motl 2009; Huang and Dong 2012). Ideally, the current results should be replicated to verify if the validity of the Czech version of Body Appreciation Scale-2 holds when correlated with the full measures. Another limitation lies in the fact that test-retest reliability was not examined. Nor were predictive and incremental validity tested. It would be beneficial to study whether the Czech version of Body Appreciation Scale-2 is prospectively associated with the unique changes in well-being above the explanatory power of the evaluative dimension of body image (e.g., body satisfaction). The investigation of incremental validity was performed by Tylka and Wood-Barcalow (2015a) for the original scale; their results supported the incremental validity of the BAS-2 and the theoretical foundations of positive body image. The same could be examined for the Czech version to provide additional evidence of validity. Concerning the directions for future research, it would be beneficial to investigate the psychometric properties of the (Czech) BAS-2 among older people, who have not yet been sufficiently targeted in prior research (e.g., Junqueira et al. 2019; Swami et al. 2019; Tylka and Wood-Barcalow 2015a). Evidence from the older population would be helpful and may facilitate research on positive body image among older adults.

## Conclusions

Body Appreciation Scale-2 (Tylka and Wood-Barcalow 2015a) has recently started to be used routinely. Our research contributed to the prior studies on the psychometric qualities of the original and translated versions (e.g., Alleva et al. 2016; Lemoine et al. 2018; Razmus and Razmus 2017) by examining the scale’s validity among Czech adolescents. The results documented optimal internal consistency, the unidimensional factor structure, metric and scalar gender and age measurement invariance, and the expected associations with the related constructs. This supports the validity of the BAS-2 among Czech adolescents. The exploration of the gender differences showed that body appreciation is more strongly related to body satisfaction, media-ideal internalization, and depression among girls than for boys. This suggests the potential accentuated role of body appreciation in the well-being of adolescent girls. Overall, the BAS-2 can be recommended for the assessment of body appreciation among Czech adolescents, and for the comparison of body appreciation among girls and boys, and younger and older adolescents.

### Supplementary Information

**Additional file 1.** **Supplemental online material**. Document with the translated scales and additional analyses.

**Additional file 2.** Analytical script (R).

## Data Availability

The datasets used and/or analyzed in the current study are freely available at: osf.io/ybkvn.

## Abbreviations

BAS-2: Body appreciation Scale-2
CFA: Confirmatory factor analysis
SEM: Structural equation modeling
MG-SEM: Multigroup structural equation modeling
HTMT: Heterotrait-monotrait ratio of correlations
CAWI: Computer-assisted web interviewing
EDI-3: Eating disorder inventory-3
ASI-R: Appearance schemas inventory-revised
MLR: Robust maximum likelihood
FIML: Full information maximum likelihood
AVE: Average variance extracted
