# Supplementary material for: The validity of the Czech version of Body Appreciation Scale-2 for adolescents
Source: J Eat Disord. 2023 Oct 5;11:176. doi: 10.1186/s40337-023-00897-7 (PMC10557209; doi:10.1186/s40337-023-00897-7)
Supplement: Supplementary file 1 — Additional file 1. Supplemental online material. Document with the translated scales and additional analyses. [file 40337_2023_897_MOESM1_ESM.docx]

**Supplemental online material**

The Czech translation of Body Appreciation Scale-2 used in this study:

Teď si prosím přečti tato tvrzení a uveď, jak často si myslíš nebo zažíváš následující věci.

(1) Nikdy, (2) Málokdy, (3) Občas, (4) Často, (5) Téměř vždy

1. Vážím si svého těla

2. Cítím se ohledně svého těla dobře

3. Myslím, že mé tělo má alespoň nějaké dobré vlastnosti

4. Mám ke svému tělu pozitivní postoj

5. Věnuji pozornost tomu, co mé tělo potřebuje

6. Mám své tělo rád/a

7. Cením si jedinečných vlastností, které mé tělo má

8. Na mém chování jde vidět, že mám dobrý vztah ke svému tělu

9. Ve svém těle se cítím dobře

10. Myslím, že jsem hezký/á, i když vypadám jinak než atraktivní lidi na internetu (např. modelové/modelky, herci/herečky)

| Item | Girls | | | Boys | | | Total sample | | |
| --- | --- | --- | --- | --- | --- | --- | --- | --- | --- |
|  | M (SD) | λ | ITC | M (SD) | λ | ITC | M (SD) | λ | ITC |
| 1. I respect my body | 3.5 (1.1) | .71 | .74 | 3.6 (1.0) | .76 | .70 | 3.6 (1.0) | .73 | .72 |
| 2. I feel good about my body | 3.4 (1.1) | .77 | .83 | 3.7 (1.0) | .86 | .76 | 3.6 (1.0) | .82 | .80 |
| 3. I feel that my body has at least some good qualities | 3.8 (0.9) | .72 | .75 | 3.9 (0.9) | .77 | .69 | 3.9 (0.9) | .74 | .72 |
| 4. I take a positive attitude towards my body | 3.6 (1.1) | .81 | .84 | 3.9 (1.0) | .86 | .78 | 3.7 (1.0) | .84 | .82 |
| 5. I am attentive to my body’s needs | 3.5 (1.0) | .58 | .52 | 3.4 (1.0) | .50 | .60 | 3.5 (1.0) | .52 | .55 |
| 6. I feel love for my body | 3.6 (1.1) | .80 | .85 | 3.9 (1.0) | .88 | .78 | 3.7 (1.1) | .85 | .82 |
| 7. I appreciate the different and unique characteristics of my body | 3.4 (1.1) | .71 | .73 | 3.6 (1.1) | .73 | .70 | 3.5 (1.1) | .72 | .72 |
| 8. My behavior reveals my positive attitude toward my body | 3.1 (1.2) | .66 | .76 | 3.4 (1.1) | .77 | .67 | 3.3 (1.2) | .72 | .72 |
| 9. I am comfortable in my body | 3.6 (1.1) | .76 | .81 | 4.0 (1.0) | .85 | .73 | 3.8 (1.1) | .81 | .78 |
| 10. I feel like I am beautiful even if I am different from media images of attractive people (e.g., models, actresses/actors) | 3.4 (1.1) | .63 | .70 | 3.4 (1.1) | .72 | .61 | 3.4 (1.1) | .67 | .65 |

**Table 1.** Means, standard deviations, factor loadings, and item-total correlations from Sample 2

**Table 2.** Factor loadings for the utilized measures in the total sample and girls and boys separately

| Items | Total sample | Girls | Boys |
| --- | --- | --- | --- |
| Internalization1 | 0.754 | 0.775 | 0.716 |
| Internalization2 | 0.773 | 0.746 | 0.782 |
| Internalization3 | 0.800 | 0.823 | 0.765 |
| Internalization4 | 0.897 | 0.881 | 0.914 |
| Internalization5 | 0.665 | 0.669 | 0.650 |
| Schematicity1 | 0.762 | 0.727 | 0.762 |
| Schematicity2 | 0.800 | 0.768 | 0.803 |
| Schematicity3 | 0.762 | 0.757 | 0.731 |
| Schematicity4 | 0.667 | 0.645 | 0.652 |
| Schematicity5 | 0.630 | 0.593 | 0.667 |
| Schematicity6 | 0.732 | 0.736 | 0.717 |
| Schematicity7 | 0.745 | 0.753 | 0.727 |
| Schematicity8 | 0.719 | 0.723 | 0.692 |
| Schematicity9 | 0.600 | 0.536 | 0.680 |
| Self-esteem1 | 0.830 | 0.825 | 0.833 |
| Self-esteem2 | 0.857 | 0.880 | 0.828 |
| Self-esteem3 | 0.746 | 0.719 | 0.766 |
| Self-esteem4 | 0.638 | 0.620 | 0.650 |
| Self-esteem5 | 0.760 | 0.734 | 0.781 |
| Depression1 | 0.844 | 0.861 | 0.787 |
| Depression2 | 0.727 | 0.727 | 0.708 |
| Depression3 | 0.792 | 0.787 | 0.769 |
| Depression4 | 0.787 | 0.788 | 0.747 |
| Body satisfaction1 | 0.322 | 0.811 | 0.893 |
| Body satisfaction2 | 0.856 | 0.834 | 0.837 |
| Body satisfaction3 | 0.813 | 0.484 | 0.579 |
| Body satisfaction4 | 0.689 | 0.318 | 0.340 |
| Body satisfaction5 | 0.578 | 0.398 | 0.420 |

**Table 3**. Results for the gender invariance testing for the utilized measures.

|  | CFI | TLI | RMSEA | SRMR |
| --- | --- | --- | --- | --- |
| Internalization |  |  |  |  |
| Configural | 0.993 | 0.983 | 0.071 | 0.021 |
| Metric | 0.993 | 0.988 | 0.059 | 0.034 |
| Scalar | 0.991 | 0.989 | 0.058 | 0.036 |
| Schematicity |  |  |  |  |
| Configural | 0.970 | 0.958 | 0.071 | 0.034 |
| Metric | 0.969 | 0.963 | 0.066 | 0.047 |
| Scalar | 0.960 | 0.957 | 0.071 | 0.054 |
| Self-esteem |  |  |  |  |
| Configural | 0.975 | 0.950 | 0.111 | 0.029 |
| Metric | 0.972 | 0.960 | 0.099 | 0.041 |
| Scalar | 0.970 | 0.960 | 0.090 | 0.045 |
| Depression |  |  |  |  |
| Configural | 0.996 | 0.988 | 0.058 | 0.013 |
| Metric | 0.999 | 0.998 | 0.022 | 0.015 |
| Scalar | 0.987 | 0.985 | 0.066 | 0.035 |
| Body satisfaction |  |  |  |  |
| Configural | 0.919 | 0.838 | 0.162 | 0.059 |
| Metric | 0.920 | 0.885 | 0.136 | 0.059 |
| Scalar | 0.910 | 0.900 | 0.127 | 0.068 |

**Czech translation of the other used scales**

1. Media-ideal internalization

Chtěl/a bych, aby moje postava vypadala jako ta jejich

Chtěl/a bych změnit něco na svém vzhledu

Chtěl/a bych zhubnout nebo naopak přibrat

Chtěl/a bych změnit to, jak vypadá moje postava

Chtěl/a bych vypadat stejně jako ony (version for girls)/oni (version for boys)

1. Self-esteem

Jsem se sebou vcelku spokojený/á

Mám k sobě dobrý vztah

Myslím si, že mám řadu dobrých vlastností

Jsem schopný/á dělat mnoho věcí stejně dobře jako ostatní

Věřím, že mám alespoň takovou hodnotu jako většina ostatních

1. Depression

Cítil/a jsem se nešťastně nebo posmutněle

Budoucnost se mi zdála beznadějná

Cítil/a jsem se napjatě nebo neklidně

Dělal/a jsem si hodně starostí
